# Examining within-session test-retest reliability of four bioimpedance devices among adults across a wide age range

**DOI:** 10.2478/joeb-2026-0010

**Published:** 2026-08-08

**Authors:** Kworweinski Lafontant, David H. Fukuda, Sofea Smith, Jack Livingston, Michelle Da Silva Barbera, Ngoc Linh Nhi Nguyen, Edwin Rodriguez-Cruz, Susan Kampiyil, Katie R. Hirsch, Sarah K. Fretti, Jeffrey R. Stout

**Affiliations:** Physiology of Work and Exercise Response (POWER) Lab, University of Central Florida, Orlando, FL, USA; Department of Kinesiology and Health Science, Utah State University, Logan, UT, USA; Department of Kinesiology, East Carolina University, Greenville, NC, USA; Arnold School of Public Health, University of South Carolina, Columbia, SC, USA; Clinical Applications for Rest and Exercise (CARE) Lab, University of Central Florida, Orlando, FL, USA

**Keywords:** Bioimpedance, current, voltage

## Abstract

Bioimpedance is increasingly growing in clinical utility for within-session monitoring; however, many studies neglect to report the test-retest reliability of their devices. The purpose of this study was to examine the within-session test-retest reliability of whole-body bioelectrical variables measured by four common bioimpedance devices among adults. Ninety-six adults (age = 46.1 ± 21.7yrs, female = 63.5%, BMI = 26.4 ± 5.2 kg/m^2^) were tested in the morning-time on each device after providing a urine sample to confirm euhydration in addition to 10 minutes of passive rest in each device’s respective testing position (standing or supine). Each device assessed whole-body impedance, resistance, reactance, and phase angle in duplicate with approximately 10 seconds between trials; bioelectrical variables were extracted at 5, 50, 250, 500, and 1000 kHz as available from each device. Reliability was assessed using intraclass correlation coefficients (ICC_2,1_) and coefficients of variation (CV). At 5, 50, and 250 kHz, reliability across devices and measures was excellent (ICC_2,1_ > 0.90, CV = 0.78 ± 0.55%). Precision at 500 kHz for PhA (CV = 5.41%) and Xc (CV = 4.9%) was low and approaching low, respectively. Measures of Xc and PhA at higher frequencies (500 and 1000 kHz) may be less precise, but further research is needed to confirm, particularly with 1000 kHz where negative Xc values may limit the interpretability of CVs. Clinicians, researchers, and practitioners using these devices for within-session monitoring of raw bioelectrical variables should ideally test the reliability of their own devices but may use the data within this manuscript as a point of reference.

## Introduction

Bioimpedance has become an increasingly popular technique, commonly used to estimate body composition and hydration status in clinical and recreational settings. Encapsulating both bioelectrical impedance analysis (BIA) and bioelectrical impedance spectroscopy (BIS), bio-impedance assesses the bioelectrical properties in the body using a few (typically < 10 for BIA) or many (typically > 100 for BIS) frequencies to estimate body composition. However, there has been a recent uptick of interest in analyzing the raw bioelectrical data from bioimpedance rather than utilizing them to estimate body composition [1,2,3]. From bioelectrical data, clinicians can examine the overall opposition of cells towards electrical currents (impedance; Z), the resistance of cells to electrical currents from the total fluid and fluid distribution (resistance; R), the dielectric properties of cell membranes (reactance; Xc), and the overall cell membrane integrity (phase angle; PhA) [4, 5]. Particularly with PhA, as a single number that accounts for Z, R, and Xc (PhA = atg(Xc/R) × (180/π)), a host of previous research has demonstrated great clinical utility, with PhA being applied as a clinical marker of sarcopenia and a prognostic sign of malnutrition [2, 6, 7]. Yet, similar to the estimations of body composition stemming from bioimpedance, there are concerns regarding the reliability of bioelectrical data.

Previous research has demonstrated challenges to inter-device reliability, with small but statistically significant differences in Z, R, Xc, and PhA being observed between devices [8]. These differences are likely due to several factors, including differences in posture and electrodes [9,10,11,12]. Importantly, these inter-device differences do not negate the clinical practicality of these devices, as standardized approaches to bioimpedance measurements can allow clinicians and practitioners to mitigate potential sources of technical error and make informed comparisons between BIA/BIS devices [13]. However, the potential impact of test-retest reliability within each device may also impact interpretations of bioelectrical data. While test-retest reliability can be device-specific and should be assessed by each device user prior to assessment, it is critical that references for test-retest reliability within each device model are readily available.

Despite the popular use of bioimpedance, many studies neglect to report the test-retest reliability of their devices. Even within studies that do report test-retest reliability, it is typically done briefly within the methods section or supplemental files of a manuscript that does not focus on test-retest reliability [14, 15], making it difficult for clinicians and researchers to locate a point of reference for their device’s reliability. Furthermore, differences in methodology have made comparison of results between studies difficult, as some studies introduced potential between-day differences [16], examined segmental reliability rather than whole-body [17], allowed participants to change postures between assessments [18], or assessed reliability against a known circuit board rather than a human participant [8]. Even among human-participant studies, there is potential variation of bioimpedance reliability due to age-related differences in skin conductance and hydration among older adults [19, 20], demonstrating a need to determine reliability for bioimpedance devices with both younger and older adults.

While these previous studies have provided us with useful insights, there remains a need for research investigating within-session test-retest reliability of whole-body bioimpedance measures *in vivo* under recommended testing controls for clinical practice. Bioimpedance continues to be used for within-session monitoring of fluid status/hemodynamics and has recently demonstrated potential utility for within-session monitoring of nutrient intake/status [21,22,23,24], making within-session reliability an important measure. Knowledge of the within-session test-retest reliability of common bioimpedance devices can serve as a reference for clinicians and practitioners that seek to compare the reliability they observe from their own devices to a standard, and the reliability information may aid interpretations of within-session changes in raw bioelectrical data using these devices. Therefore, the purpose of this study was to examine the within-session test-retest reliability of whole-body bioelectrical variables measured by four common bioimpedance devices among a broad age range of adults. We hypothesized that all four devices would exhibit excellent reliability (ICC_2,1_ ≥ 0.90, CV ≤ 5%) across all whole-body bioelectrical variables.

## Materials and methods

### Informed consent

Written informed consent has been obtained from all individuals included in this study.

### Ethical approval

The research related to human use has been complied with all relevant national regulations, institutional policies and in accordance with the tenets of the Helsinki Declaration and has been approved by the University of Central Florida Institutional Review Board (ID: STUDY00007777).

### Study Design

We utilized a randomized, counter-balanced cross-sectional design with 96 total participants, recruited between May 2025 and October 2025. Participants were recruited using social media postings, fliers posted at local gyms and community centers, and word-of-mouth around the greater Orlando, FL, metropolitan area. Participants were included in this study if they were ≥ 18 years of age, free from implanted pacemakers/defibrillators, not pregnant, not missing limbs, and not using any prosthetic devices.

**Figure 1.**
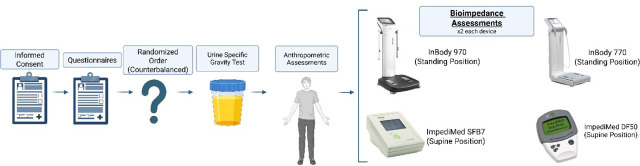
Flow of study events.

All study visits were completed in the morning (7:30 – 11:30 am) in a climate-controlled environment (Barometric Pressure = 760.4 ± 2.4 mmHg, Temperature = 23.3 ± 0.5 °C, Humidity = 45.8 ± 1.2%). Figure 1 illustrates the flow of study events. Participants were instructed to arrive to their study visit wearing light, athletic clothing, with no hair on their right hand/wrist and right foot/ankle, dry fasted for at least 3 hours (i.e., no food, water, or beverages), free from caffeine for at least 12 hours, and free from alcohol as well as strenuous physical activity/exercise for at least 24 hours. The duration of the dry fast was based on clinical feasibility given prior recommendations [4]. Participants were also instructed to remove all jewelry. After obtaining informed consent, participants self-reported age, sex, race/ethnicity, and hand dominance, which was recorded on a data collection sheet by a trained research assistant.

Upon completing the questionnaires, participants were provided with a urine sample cup and were given instructions to collect a mid-stream urine sample while voiding their bladder. The mid-stream urine sample was analyzed for urine specific gravity (USG) using a MISCO PA202x Palm Abbe digital refractometer (MISCO Refractometer, Solon, OH, USA) to ensure adequate hydration (USG < 1.030). If participants were inadequately hydrated, they were rescheduled to complete the bioimpedance assessments on another day following rehydration. After ensuring adequate hydration, we utilized a digital physician scale and stadiometer (Health-O-Meter^TM^, Model 402KL, McCook, IL, USA) to assess height and body mass. Participants then completed the bioimpedance assessments in their randomized order between all four devices. Assessments were nested into either standing or supine assessments, with devices inside each nest being randomized in addition to the randomized order of the nests. All bioimpedance devices were separated by at least 15 feet (4.57 meters) to mitigate any potential for inter-device interference.

### Standing Bioimpedance Assessments

Prior to completing standing bioimpedance assessments, participants rested in the standing position for 10 minutes. Following the standing rest, participants completed assessments using the InBody 770 (InBody BWA, Audubon, PA, USA) and InBody 970 (InBody BWA, Audubon, PA, USA). Both devices are phase sensitive, direct segmental, multi-frequency BIA devices. The InBody 770 uses 1, 5, 50, 250, 500, and 1000 kHz frequencies, while the InBody 970 uses the same frequencies as well as 2000 and 3000 kHz. Both devices contain an internal calibration circuit that automatically checks calibration at start-up prior to each assessment. For both devices, participants were given an InBody Tissue (InBody BWA, Audubon, PA, USA) to wipe the palms/fingertips and foot soles immediately prior to stepping onto the BIA device on pre-marked touch-type sensors, holding onto the touch-type handle electrodes with each hand, and following all on-screen instructions. Participants completed two tests consecutively (with a 10-second break between tests) on one device before using the other device, and participants were instructed to remain silent and motionless during each test. During the 10-second breaks, participants had to step down from each device and step back up to reset the device and allow for a second test to be completed. A unique profile was created for each test to account for any potential influence of device memory [25]. Z at 5, 50, 250, 500, and 1000 kHz was extracted directly from both devices. Neither the InBody 970 nor 770 reports R, Xc, and PhA at 500 and 1000 kHz, so Xc and PhA were extracted at only 5, 50, and 250 kHz. R is not directly reported by the InBody devices, so it was calculated at 5, 50, and 250 kHz as the square rooted difference of Z^2^ and Xc^2^ [26].

### Supine Bioimpedance Assessments

**Figure 2.**
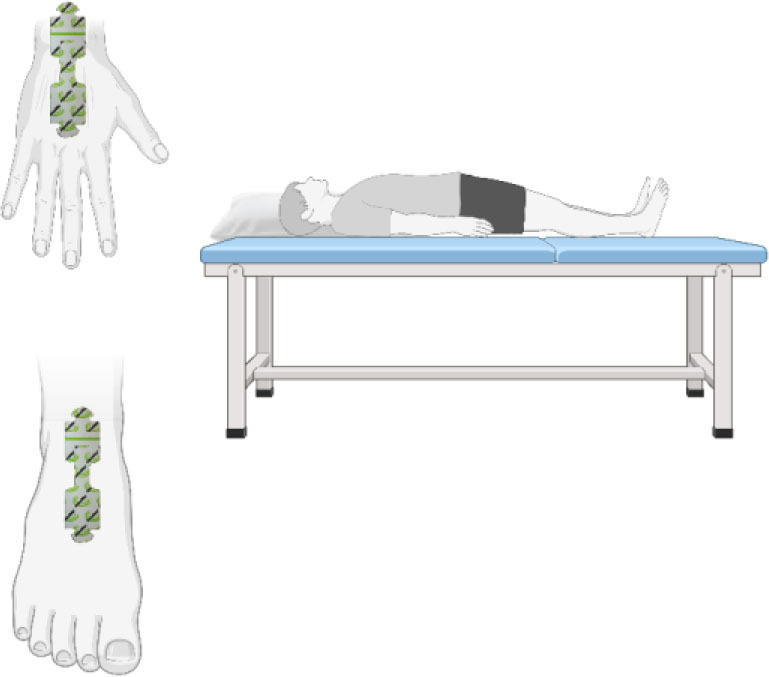
Placement of dual-tab electrodes on right hand and foot. Participants were positioned supine on a padded table with a pillow underneath their head and their arms and legs abducted.

Prior to completing supine bioimpedance assessments, participants rested in the supine position for 10 minutes on a padded table with a pillow underneath their head, and their arms/legs abducted approximately 30 degrees to minimize thigh/torso contact. During their 10 minutes of rest, we utilized a 70% isopropyl alcohol wipe to prepare the skin on the dorsal side of the right hand/wrist and right foot/ankle. Then, we placed ImpediMed solid-gelled dual-tab electrodes (ImpediMed Inc., Carlsbad, CA, USA; item reference number: 292-BCE; length = 75 mm, width = 23mm) on the dorsal side of the right hand/wrist and right foot/ankle (Figure 2). The proximal ends of hand and foot electrodes were placed medially to the radial and ulnar styloid process and medially to the malleoli, respectively. Following the supine rest, participants completed assessments using the ImpediMed SFB7 (ImpediMed Inc., Carlsbad, CA, USA) and ImpediMed DF50 (ImpediMed Inc., Carlsbad, CA, USA). The SFB7 is a phase sensitive BIS device, utilizing 256 frequencies ranging from 4 – 1000 kHz. The DF50 is a single-frequency (50 kHz) BIA device. Both devices were checked for calibration each morning prior to assessments using a manufacturer supplied test-cell. Participants completed two tests consecutively (with a 10 second break between tests without repositioning electrodes or movement of the body) on one device before using the other device, and participants were instructed to remain silent and motionless during each test. The same electrodes were used for both devices and then discarded, with a new set of electrodes used for each participant. For the SFB7, a unique profile was created for each test to account for any potential influence of device memory [25]; the DF50 does not save user profiles.

From the DF50, Z, R, Xc, and PhA were extracted directly at 50 kHz. For the SFB7, we utilized the manufacturer-provided BioImp software version 5.5.0.1 (ImpediMed Inc., Carlsbad, CA, USA) to process bioelectrical variables using a 5 – 1000 kHz frequency range and automatic rejection limits to remove outlier data points based on non-iterative curvilinear Cole-Cole modeling. After processing, we extracted measured Z, R, Xc, and PhA at 5, 50, 250, 500, and 1000 kHz from the BioImp software.

### Statistical Analysis

All statistical analyses were completed using Jamovi version 2.7.12 and its “SimplyAgree” package [27,28,29]. The assumption of normality was confirmed using a Kolmogorov-Smirnov test and visual inspection of histograms. Intra-class correlation coefficients (ICC_2,1_) with 95% confidence intervals (CI), coefficients of variation (CV), and standard error of the measurement (SEM) were calculated to assess intra-device reliability and precision on bioelectrical variables (Z, R, Xc, and PhA) at 5, 50, 250, 500, and 1000 kHz.

Additionally, we used SEM values to calculate minimal detectable change (MDC) for each outcome variable (MDC = SEM × 1.96 × √2), and paired t-tests to compare measures between trials at each frequency and report mean absolute differences (MAD). The DF50 only utilized a single 50 kHz frequency, so it was omitted from analyses for all other frequencies. While not the primary focus on this study, we also assessed segmental within-session intra-device reliability of raw bioelectrical variables at 5, 50, 250, 500, and 1000 kHz for the InBody 970 and 770, with results provided in Supplemental File 1. Given the wide range of sampled ages, we conducted simple slope moderation analyses on whole-body variables to determine if age was a significant moderator of the relationship between each device’s two trials using ±1 standard deviation (SD) in age. Similarly, we conducted linear regression moderation analyses with sex as a factor on whole-body variables to determine if sex moderated the relationship between each device’s two trials. The threshold for statistical significance was set at p < 0.05.

## Results

Table 1 provides demographic information for participants. All participants were well hydrated at the time of assessments (USG = 1.015 ± 0.006). Table 2 details the measured bioelectrical data for each device at each included frequency, averaged between both trials.

**Table 1.** Participant demographics (N = 96).

Age (years)	46.1 ± 21.7
	(19 – 81)

Weight (kg)	73.7 ± 14.9
	(46.5 – 111.2)

Height (cm)	167.0 ± 9.4
	(123.0 – 190.0)

BMI (kg/m^2^)	26.4 ± 5.2
	(17.1 – 47.1)

Sex	M: 35 (36.5%)
	F: 61 (63.5%)

Ethnicity	H: 16 (16.7%)
	NH: 80 (83.3%)

Race	A: 7 (7.3%)
	B: 6 (6.3%)
	L: 6 (6.3%)
	M: 3 (3.1%)
	W: 70 (72.8%)
	O: 4 (4.2%)

**Note.** BMI = body mass index, M = male, F = female, H = Hispanic, NH = non-Hispanic, A = Asian, B = Black, L = Latino, M = Middle Eastern/North African, W = White, O = other. Data are presented as mean ± standard deviation, n (%), or range.

**Table 2.** Whole-body bioimpedance by device at various frequencies.

***InBody 970***	**Z (Ω)**	**Xc (Ω)**	**R (Ω)**	**PhA (°)**
*5kHz*	675.8 ± 95.4	29.0 ± 6.1	675.2 ± 95.4	2.48 ± 0.53
	(496.6 – 915.7)	(16.1 – 46.7)	(496.2 – 914.9)	(1.52 – 3.94)

*50kHz*	600.1 ± 90.6	58.0 ± 10.3	597.1 ± 90.6	5.60 ± 0.97
	(415.9 – 821.5)	(34.9 – 83.1)	(411.9 – 818.0)	(3.74 – 7.84)

*250kHz*	541.8 ± 84.9	44.8 ± 6.7	539.9 ± 84.7	4.78 ± 0.54
	(361.6 – 747.4)	(29.7 – 60.3)	(359.6 – 745.6)	(3.81 – 6.00)

*500kHz*	524.4 ± 82.6	-	-	-
	(348.0 – 727.6)			

*1000kHz*	509.6 ± 80.5	-	-	-
	(337.3 – 709.6)			

***InBody 770***	**Z (Ω)**	**Xc (Ω)**	**R (Ω)**	**PhA (°)**

*5kHz*	681.1 ± 97.0	30.8 ± 6.5	680.3 ± 97.0	2.61 ± 0.53
	(495.2 – 924.3)	(18.1 – 51.3)	(494.8 – 923.6)	(1.67 – 4.04)

*50kHz*	604.3 ± 91.0	59.2 ± 10.4	601.2 ± 91.0	5.68 ± 0.98
	(418.2 – 828.2)	(35.5 – 85.8)	(414.1 – 824.6)	(3.86 – 7.95)

*250kHz*	543.6 ± 84.8	46.0 ± 7.0	542.5 ± 84.9	4.88 ± 0.52
	(360.4 – 747.2)	(30.8 – 60.6)	(361.0 – 746.8)	(3.92 – 6.10)

*500kHz*	526.0 ± 82.7	-	-	-
	(345.6 – 723.6)			

*1000kHz*	513.5 ± 80.8	-	-	-
	(334.9 – 709.9)			

***ImpediMed DF50***	**Z (Ω)**	**Xc (Ω)**	**R (Ω)**	**PhA (°)**

*50kHz*	545.4 ± 85.4	59.5 ± 10.9	542.1 ± 85.4	6.34 ± 1.15
	(372.0 – 745.1)	(34.5 – 86.8)	(367.7 – 741.1)	(4.00 – 9.25)

***ImpediMed SFB7***	**Z (Ω)**	**Xc (Ω)**	**R (Ω)**	**PhA (°)**

*5kHz*	627.6 ± 91.9	30.6 ± 6.9	626.8 ± 91.8	2.82 ± 0.62
	(455.8 – 850.9)	(16.0 – 47.5)	(455.4 – 850.0)	(1.80 – 4.65)

*50kHz*	548.3 ± 86.0	57.1 ± 10.6	545.5 ± 85.9	6.03 ± 1.11
	(374.5 – 749.5)	(32.4 – 79.2)	(370.5 – 745.7)	(3.90 – 8.50)

*250kHz*	490.1 ± 80.0	32.2 ± 5.7	489.0 ± 80.0	3.80 ± 0.58
	(321.8 – 675.7)	(17.3 – 42.0)	(320.6 – 674.5)	(2.50 – 5.10)

*500kHz*	477.1 ± 78.2	16.0 ± 5.1	476.8 ± 78.2	1.94 ± 0.59
	(311.5 – 659.8)	(5.0 – 29.2)	(311.2 – 659.2)	(0.50 – 3.60)

*1000kHz*	475.4 ± 78.2	−1.9 ± 8.9	475.3 ± 78.1	−0.23 ± 1.06
	(310.3 – 655.5)	(−24.7 – 20.8)	(310.3 – 655.3)	(−2.50 – 2.70)

**Note.** Z = impedance, Xc = reactance, R = resistance, PhA = phase angle. Data are presented as mean ± standard deviation and (range).

Whole-body intra-device reliability at 5 kHz (Table 3), 50 kHz (Table 4), 250 kHz (Table 5), 500 kHz (Table 6), and 1000 kHz (Table 7) are listed below. At 500 kHz, only PhA from the SFB7 device exceeded the prespecified CV threshold (5.41%). At 1000 kHz, CVs for Xc and PhA from the SFB7 were provided for completeness and may not be interpretable given the observed negative values and their impact on CV (Table 2).

**Table 3.** Whole-body within-session intra-device reliability at 5 kHz.

	**Z**	**Xc**	**R**	**PhA**
***InBody 970***				
*ICC_2,1_*	0.997 (0.993, 0.998)	0.989 (0.964, 0.995)	0.997 (0.993, 0.998)	0.993 (0.985, 0.996)
*CV*	0.66%	1.75%	0.66%	1.50%
*SEM*	4.44Ω	0.51Ω	4.44Ω	0.04°
*MDC*	12.31Ω	1.41Ω	12.31Ω	0.11°
*MAD*	3.88Ω	0.56Ω	3.85Ω	0.03°
*p-value*	< 0.001	< 0.001	< 0.001	< 0.001

***InBody 770***				
*ICC_2,1_*	0.999 (0.997, 0.999)	0.992 (0.987, 0.995)	0.999 (0.997, 0.999)	0.993 (0.990, 0.995)
*CV*	0.39%	1.79%	0.39%	1.64%
*SEM*	2.65Ω	0.55Ω	2.65Ω	0.04°
*MDC*	7.35Ω	1.52Ω	7.35Ω	0.11°
*MAD*	2.49Ω	0.32Ω	2.48Ω	0.02°
*p-value*	< 0.001	< 0.001	< 0.001	0.01

***SFB7***				
*ICC_2,1_*	1.00 (1.00, 1.00)	0.999 (0.999, 1.00)	1.00 (1.00, 1.00)	0.998 (0.997, 0.998)
*CV*	0.08%	0.53%	0.08%	1.05%
*SEM*	0.49Ω	0.16Ω	0.49Ω	0.03°
*MDC*	1.36Ω	0.44Ω	1.36Ω	0.08°
*MAD*	0.14Ω	0.06Ω	0.13Ω	0.005°
*p-value*	0.06	0.01	0.07	0.23

**Note.** Z = impedance, Xc = reactance, R = resistance, PhA = phase angle, ICC = intraclass correlation coefficient, CV = coefficient of variation, SEM = standard error of the measurement, MAD = mean absolute difference. P-values are presented for pairwise comparisons between trials one and two for each variable and correspond with MAD but are not interpreted on their own as direct measures of agreement. The threshold for statistical significance set at p < 0.05.

**Table 4.** Whole-body within-session intra-device reliability at 50 kHz.

	**Z**	**Xc**	**R**	**PhA**
***InBody 970***				
*ICC_2,1_*	0.999 (0.997, 0.999)	0.997 (0.994, 0.999)	0.999 (0.997, 0.999)	0.998 (0.997, 0.999)
*CV*	0.48%	0.77%	0.48%	0.77%
*SEM*	2.86Ω	0.45Ω	2.86Ω	0.04°
*MDC*	7.93Ω	1.25Ω	7.93Ω	0.11°
*MAD*	2.49Ω	0.38Ω	2.46Ω	0.01°
*p-value*	< 0.001	< 0.001	< 0.001	0.07

***InBody 770***				
*ICC_2,1_*	0.999 (0.998, 1.00)	0.998 (0.996, 0.998)	0.999 (0.998, 1.00)	0.998 (0.997, 0.998)
*CV*	0.38%	0.87%	0.38%	0.86%
*SEM*	2.30Ω	0.52Ω	2.30Ω	0.05°
*MDC*	6.38Ω	1.44Ω	6.38Ω	0.14°
*MAD*	1.82Ω	0.13Ω	1.81Ω	0.006°
*p-value*	< 0.001	0.09	< 0.001	0.38

***DF50***				
*ICC_2,1_*	0.999 (0.999, 1.00)	0.996 (0.995, 0.997)	0.999 (0.999, 1.00)	0.986 (0.981, 0.990)
*CV*	0.35%	1.08%	0.37%	2.15%
*SEM*	1.91Ω	0.64Ω	2.00Ω	0.14°
*MDC*	5.29Ω	1.77Ω	5.54Ω	0.39°
*MAD*	0.51Ω	0.08Ω	0.48Ω	0.01°
*p-value*	0.07	0.39	0.10	0.53

***SFB7***				
*ICC_2,1_*	0.999 (0.999, 0.999)	1.00 (1.00, 1.00)	1.00 (1.00, 1.00)	0.996 (0.995, 0.997)
*CV*	0.45%	0.30%	0.07%	1.10%
*SEM*	2.46Ω	0.17Ω	0.39Ω	0.07°
*MDC*	6.82Ω	0.47Ω	1.08Ω	0.19°
*MAD*	0.63Ω	0.05Ω	0.13Ω	0.01°
*p-value*	0.08	0.048	0.02	0.22

**Note.** Z = impedance, Xc = reactance, R = resistance, PhA = phase angle, ICC = intraclass correlation coefficient, CV = coefficient of variation, SEM = standard error of the measurement, MAD = mean absolute difference. P-values are presented for pairwise comparisons between trials one and two for each variable and correspond with MAD but are not interpreted on their own as direct measures of agreement. The threshold for statistical significance set at p < 0.05.

**Table 5.** Whole-body within-session intra-device reliability at 250 kHz.

	**Z**	**Xc**	**R**	**PhA**
***InBody 970***				
*ICC_2,1_*	0.999 (0.997, 0.999)	0.995 (0.993, 0.997)	0.999 (0.997, 0.999)	0.992 (0.988, 0.994)
*CV*	0.51%	1.02%	0.51%	1.01%
*SEM*	2.77Ω	0.46Ω	2.77Ω	0.05°
*MDC*	7.68Ω	1.28Ω	7.68Ω	0.14°
*MAD*	2.29Ω	0.09Ω	2.29Ω	0.01°
*p-value*	< 0.001	0.17	< 0.001	0.09

***InBody 770***				
*ICC_2,1_*	0.999 (0.998, 0.999)	0.990 (0.986, 0.993)	0.999 (0.998, 0.999)	0.980 (0.972, 0.986)
*CV*	0.42%	1.51%	0.43%	1.46%
*SEM*	2.30Ω	0.70Ω	2.31Ω	0.07°
*MDC*	6.38Ω	1.94Ω	6.40Ω	0.19°
*MAD*	1.76Ω	0.12Ω	1.77Ω	0.03°
*p-value*	< 0.001	0.22	< 0.001	0.01

***SFB7***				
*ICC_2,1_*	1.00 (1.00, 1.00)	0.995 (0.992, 0.996)	1.00 (1.00, 1.00)	0.990 (0.985, 0.993)
*CV*	0.13%	1.31%	0.12%	1.57%
*SEM*	0.63Ω	0.42Ω	0.61Ω	0.06°
*MDC*	1.75Ω	1.16Ω	1.69Ω	0.17°
*MAD*	0.08Ω	0.003Ω	0.08Ω	0.006°
*p-value*	0.37	0.96	0.37	0.47

**Note.** Z = impedance, Xc = reactance, R = resistance, PhA = phase angle, ICC = intraclass correlation coefficient, CV = coefficient of variation, SEM = standard error of the measurement, MAD = mean absolute difference. P-values are presented for pairwise comparisons between trials one and two for each variable and correspond with MAD but are not interpreted on their own as direct measures of agreement. The threshold for statistical significance set at p < 0.05.

**Table 6.** Whole-body within-session intra-device reliability at 500 kHz.

	**Z**	**Xc**	**R**	**PhA**
***InBody 970***		-	-	-
*ICC_2,1_*	0.999 (0.997, 0.999)			
*CV*	0.53%			
*SEM*	2.78Ω			
*MDC*	7.71Ω			
*MAD*	2.27Ω			
*p-value*	< 0.001			

***InBody 770***		-	-	-
*ICC_2,1_*	0.999 (0.998, 0.999)			
*CV*	0.44%			
*SEM*	2.30Ω			
*MDC*	6.38Ω			
*MAD*	1.80Ω			
*p-value*	< 0.001			

***SFB7***				
*ICC_2,1_*	1.00 (1.00, 1.00)	0.976 (0.967, 0.983)	1.00 (1.00, 1.00)	0.968 (0.956, 0.977)
*CV*	0.07%	4.9%	0.07%	5.41%
*SEM*	0.32Ω	0.78Ω	0.33Ω	0.10°
*MDC*	0.89Ω	2.16Ω	0.91Ω	0.28°
*MAD*	0.25Ω	0.17Ω	0.25Ω	0.02°
*p-value*	< 0.001	0.15	< 0.001	0.22

**Note.** Z = impedance, Xc = reactance, R = resistance, PhA = phase angle, ICC = intraclass correlation coefficient, CV = coefficient of variation, SEM = standard error of the measurement, MAD = mean absolute difference. P-values are presented for pairwise comparisons between trials one and two for each variable and correspond with MAD but are not interpreted on their own as direct measures of agreement. The threshold for statistical significance set at p < 0.05.

**Table 7.** Whole-body within-session intra-device reliability at 1000 kHz.

	**Z**	**Xc**	**R**	**PhA**
***InBody 970***		-	-	-
*ICC_2,1_*	0.999 (0.997, 0.999)			
*CV*	0.51%			
*SEM*	2.62Ω			
*MDC*	7.26Ω			
*MAD*	2.14Ω			
*p-value*	< 0.001			

***InBody 770***		-	-	-
*ICC_2,1_*	0.999 (0.998, 0.999)			
*CV*	0.46%			
*SEM*	2.34Ω			
*MDC*	6.49Ω			
*MAD*	1.85Ω			
*p-value*	< 0.001			

***SFB7***				
*ICC_2,1_*	1.00 (1.00, 1.00)	0.968 (0.955, 0.977)	1.00 (1.00, 1.00)	0.961 (0.945, 0.972)
*CV*	0.14%	−83.39%	0.14%	−92.05%
*SEM*	0.66Ω	1.16Ω	0.65Ω	0.21°
*MDC*	1.83Ω	3.22Ω	1.80Ω	0.58°
*MAD*	0.68Ω	0.13Ω	0.66Ω	0.01°
*p-value*	< 0.001	0.60	< 0.001	0.79

**Note.** Z = impedance, Xc = reactance, R = resistance, PhA = phase angle, ICC = intraclass correlation coefficient, CV = coefficient of variation, SEM = standard error of the measurement, MAD = mean absolute difference. CV values for SFB7 Xc and PhA at 1000kHz are shown for completeness but may not be interpretable as the corresponding mean values were near zero or negative. P-values are presented for pairwise comparisons between trials one and two for each variable and correspond with MAD but are not interpreted on their own as direct measures of agreement. The threshold for statistical significance set at p < 0.05.

Instances of statistically significant moderation effects are listed in Table 8, with simple slope figures provided in Supplemental File 1. A statistically significant moderating effect of age was observed for whole-body PhA at 250 kHz between trials with the InBody 770 (p = 0.04), for whole-body Z and R at 50 kHz between trials with the DF50 (p = 0.04), for whole-body Xc at 50 kHz between trials with the SFB7 (p = 0.02), and for whole-body Z and R at 500 kHz (p = 0.02 to 0.03) and 1000 kHz (p = 0.01) between trials with the SFB7. Regarding sex, no statistically significant moderation effects were observed.

**Table 8.** Simple slope moderation analysis results for age.

	**Condition**	**B**	**95% CI**	**p-value**
*InBody 770*	−1SD Age	1.019	0.959, 1.079	< 0.001
*250kHz – PhA*	+1SD Age	0.927	0.869, 0.984	< 0.001
	Average	0.973	0.934, 1.011	< 0.001

*ImpediMed DF50*	−1SD Age	0.997	0.989, 1.010	< 0.001
*50kHz – Z*	+1SD Age	1.010	1.001, 1.020	< 0.001
	Average	1.004	0.998, 1.010	< 0.001

*ImpediMed DF50*	−1SD Age	0.998	0.988, 1.007	< 0.001
*50kHz – R*	+1SD Age	1.011	1.002, 1.020	< 0.001
	Average	1.004	0.998, 1.011	< 0.001

*ImpediMed SFB7*	−1SD Age	0.988	0.981, 0.995	< 0.001
*50kHz – Xc*	+1SD Age	1.000	0.993, 1.007	< 0.001
	Average	0.994	0.990, 0.999	< 0.001

*ImpediMed SFB7*	−1SD Age	1.001	1.000, 1.001	< 0.001
*500kHz – Z*	+1SD Age	0.999	0.997, 1.000	< 0.001
	Average	1.000	0.999, 1.001	< 0.001

*ImpediMed SFB7*	−1SD Age	1.001	1.000, 1.001	< 0.001
*500kHz – R*	+1SD Age	0.999	0.997, 1.000	< 0.001
	Average	1.000	0.999, 1.001	< 0.001

*ImpediMed SFB7*	−1SD Age	1.001	0.997, 1.004	< 0.001
*1000kHz - Z*	+1SD Age	0.995	0.991, 0.998	< 0.001
	Average	0.998	0.995, 1.000	< 0.001

*ImpediMed SFB7*	−1SD Age	1.000	0.997, 1.004	< 0.001
*1000kHz - R*	+1SD Age	0.995	0.991, 0.998	< 0.001
	Average	0.998	0.995, 1.000	< 0.001

**Note.** Z = impedance, R = resistance, Xc = reactance, PhA = phase angle, SD = standard deviation.

## Discussion

The purpose of this study was to examine the within-session test-retest reliability of whole-body bioelectrical variables measured by four common bioimpedance devices among adults. We hypothesized that all four devices would exhibit excellent reliability (ICC_2,1_ ≥ 0.90, CV ≤ 5%) across all whole-body bioelectrical variables; this was largely supported, with all observed ICC’s > 0.90. Regarding absolute reliability, only SFB7 PhA at 500 kHz exceeded the prespecified CV threshold of 5%, not accounting for the 1000 kHz CVs for SFB7 Xc and PhA, as those CVs were not interpretable due to the corresponding means being negative and near zero. The SFB7 was the only device to provide Xc, R, and PhA measures above 250 kHz, so it is unclear if a similar phenomenon would have been observed with a different device. Furthermore, MAD values exceeded the SEM for Z and R at 1000kHz with the SFB7 device, as well as with Xc at 5kHz on the InBody 970 device. These absolute error metrics are important to interpret alongside ICCs, because the broad heterogeneity of the sample may support very high relative reliability even when small trial-level differences remain present. However, these excursions beyond the SEM were less than one ohm and may not necessarily translate to clinically significant differences. Nonetheless, the present results indicate excellent reliability and precision for the included devices at 5, 50, and 250 kHz, except for Xc and PhA in the trunk at 250 kHz. There was some potential loss of precision at 500 and 1000kHz that warrants further investigation.

Xc is a predominant contributor to the determination of PhA, as PhA is more sensitive to changes in Xc than R [3]. Therefore, the reliability of PhA is dependent on the reliability of Xc. This may explain why a loss of precision for PhA measures at 500 kHz occurred, with a CV of 4.9%. Xc reflects dielectric properties, stemming from the unequal distribution of charged ions across the membrane and the subsequent capacitive polarization. Often termed an “imaginary component” [11, 21], the nature of Xc may make it more difficult to assess reliably, and particularly at high frequencies; as the current penetrates the intracellular space at higher frequencies, capacitive reactance decreases.

Previous studies that briefly report test-retest reliability of bioimpedance devices largely do so at 50 kHz only [14], highlighting a need for more assessments of reliability at higher and lower frequencies. At 50kHz, previous studies have reported excellent reliability (ICC > 0.90) similar to what we observed in the present study across all devices included [14, 16]. However, it is also worth noting that negative Xc values were observed for several participants at 500 and 1000 kHz, which is not typically observed at lower frequencies and limits the interpretability of CV values. Attempts to interpret or use negative Xc values in this context may result in subsequent errors in ICW estimations and PhA calculations, likely increasing the risk of error in clinical decision making. Moreover, we observed instances of statistically significant differences between trials with all frequencies and with all devices except the ImpediMed DF50. We were unable to identify a pattern within these differences, given that all testing methods were consistent between devices and participants. These significant differences may further underscore some of the natural variability with bioelectrical measures; previous research utilizing bioimpedance to assess breathing patterns would suggest that small movements such as breathing may have a noticeable impact on resting bioimpedance measures and contribute partially to variability [30]. Nonetheless, future research should aim to replicate our work and further control potentially extraneous variables like respiration rate during testing.

Within the present study, we observed some instances of statistically significant moderation effects from age between measures. While statistically significant, the differences in simple slopes between −1SD and +1SD may not be practically relevant as the point estimates for all slopes remained strong and positive at b > 0.90 (Table 8; Supplemental File 1). Meaning, while these moderation analysis results indicate that reliability may differ statistically between younger and older adults, reliability likely would not change to a point that would alter conclusions from this data or clinical practice. However, clinicians should be cautious when interpreting pooled SEM and MDC values, as those values may shift when focusing on a particular age subgroup such as older adults. Nonetheless, the reliability and precision of whole-body bioelectrical variables from the devices included is strong, particularly at 5, 50, and 250 kHz.

While this study was strengthened with robust moderation analyses, a large sample size, and a randomized counter-balanced design, our work is not without limitations. Although participants ranged from 19 to 81 years, primary reliability estimates were not stratified into predefined younger and older subgroups. Rather, we performed robust moderation analyses to explore the potential moderating effect of age (Table 8) while mitigating any losses in statistical power. Participants in the present study were predominantly non-Hispanic and White; although we are unaware of any rationale for how this may impact test results, it is still important to note the discrepancy in race and ethnicity for clinicians and practitioners that wish to translate the present results into practice. Previous work by Graybeal et al. [31] has demonstrated no significant differences in raw bioelectrical variables between White and Black adults when matched for sex, age, and BMI, suggesting that race/ethnicity is not a driving factor behind potential interindividual differences in bioimpedance characteristics. Regarding methodology, participants had to step off each standing BIA device to reset it prior to the subsequent trial due to manufacturer settings. This introduces an additional potential for error that could be viewed as separate from the device itself (i.e., variance in how participants step influencing their results) or could be viewed as a part of the designed device operations based on manufacturer settings; clinicians should be aware of this and use their judgement when interpreting present results accordingly. Furthermore, regarding methodology, recommendations for the utilization of bioimpedance distinguish between research and clinical practice, as the two occasionally differ [13]. For example, fasting prior to bioimpedance assessments is recommended to be ≥ 8 hours within research settings, yet it is also acknowledged that shorter fasting windows are acceptable in clinical practice [13]. We chose to align pre-visit fasting requirements for participants with clinical practicality to improve the translation of our work into clinical practice, as research arguably should do.

Within the translation into practice, clinicians should also be cautious if they use bioimpedance devices that were not included in the present study. Differences in raw bioelectrical variables have been reported between devices [8], so reliability metrics should be ascribed only to the same make and model bioimpedance device. Even still, we maintain that best practice for clinicians would be to assess the reliability of their own devices, in the event of inter-device discrepancies within the same make and model. We designed the present testing procedures to inform within-session reliability with minimal participant movement and electrode repositioning between measures, similar to previous work by Siedler et al [25]. However, we were limited in our ability to extend the duration between each measure due to the time burden for participants that would have ensued. Results may have differed with changes in participant activity, electrode repositioning, and duration between measures. Previous work has demonstrated alterations in bioelectrical variables following passive rest in a thermoneutral environment for 5 hours, attributed to dehydration [32]. Assessments of reliability are sensitive to a multitude of variables that cannot reasonably be permuted and included into a single study design, and that limitation must be considered when applying this work. Similarly, raw bioelectrical variables are often used to estimate body composition parameters such as total body water, fat mass, and fat-free mass [33]. This study did not assess the reliability of these estimated parameters as the estimation equations used by clinicians vary according to device and population characteristics (age, sex, race/ethnicity, training status, etc.). Our decision to focus on intra-day reliability also limits the ability to translate our results to inter-day testing. Previous work by Garr Barry et al. [34] examined intra- and inter-day reliability of membrane capacitance, a similar bioelectrical impedance variable, using an ImpediMed SFB7. Their results indicated an intra-day (30-minute delay between trials) technical error of measurement of 3.43% compared to an inter-day (1–2 week delay between trials) technical error of measurement of 7.93%. Generally, stronger reliability can be expected intra-day compared to inter-day given the tighter control of potentially extraneous variables, such as diet, skin temperature, and hydration, between trials.

## Conclusion

Within-session test-retest reliability of the InBody 770, InBody 970, ImpediMed SFB7, and ImpediMed DF50 at their applicable frequencies of 5, 50, and 250 kHz appear to be excellent among adults of a wide age range, except for Xc and PhA in the trunk at 250 kHz. Measures of Xc and PhA at higher frequencies (500 and 1000 kHz) may be less precise, but further research is needed to confirm this theory. As observed Xc values at higher frequencies (such as 1000 kHz) near and potentially cross zero, CV values should be interpreted cautiously, and other reliability metrics may be preferable. Clinicians, researchers, and practitioners using these devices for within-session monitoring of raw bioelectrical variables should ideally test the reliability of their own devices but may use the data within this manuscript as a point of reference.

## Supplemental Files

Supplemental tables and figures: https://github.com/joeb-files/2026_Lafontant
